# Supplementary material for: Fitness advantage of sequential metabolic strategies emerges from community interactions in strongly fluctuating environments
Source: PLoS Comput Biol. 2026 May 12;22(5):e1014277. doi: 10.1371/journal.pcbi.1014277 (PMC13232955; doi:10.1371/journal.pcbi.1014277)
Supplement: S1 Text — Green: Same as the green line in main text Fig 2E, where pool size N=100, and all sequential species are top-smart. Red: red lines of different shades (pool size from 100 to 400, with 1:1 fraction of sequential to co-utilizing strategists) correspond to community assembly outcomes, where sequential species have random preference orders. In these simulations, since the probability of being “top-smart” is 1/nR=1/4, achieving a similar level of sequential species prevalence requires a species pool approximately 4 times larger than in our main simulations (green). Fig B Ecological effects of metabolic strategies when lags are absent. (a) Fraction of surviving sequential utilizers in each of the two scenarios: head-to-head (black) and in complex communities (green), plotted as a function of the pre-allocation fraction ϕ common to all species in the pool. In the absence of lags, the competitive performance of sequential species is significantly enhanced: compared to the results with lags (Fig 2E), their biomass fraction in head-to-head competitions exhibits as high as ∼ 5-fold increase, while the increase in assembled communities is more moderate. The gray horizontal line denotes competitive parity (biomass fraction=0.5). The black vertical line lies at ϕ=0.0615, where sequential species hold a slight advantage in communities, but a slight disadvantage in head-to-head competitions. (b) Distributions of the maximum growth rates sampled from the underlying populations shown in (c); sequential species possess greater extreme values due to their broader distribution tails, enabling them to dominate in complex communities where competition is dictated by extreme value statistics. (c) Population-level growth rate distributions of sequential (blue) and co-utilizing (red) species in the first temporal niche at ϕ=0.0615, highlighting that sequential species exhibit higher variance despite a lower mean.(d)-(e) Time-average growth rates of communities as a function of ϕ. (d) In mono [file pcbi.1014277.s001.pdf]

S1 Text for
Fitness advantage of sequential metabolic strategies emerges from community interactions in strongly fluctuating environments

Zihan Wang[1,2], Yu Fu[3], Akshit Goyal[4,*], Sergei Maslov[1,2,5*]

[1] *Department of Physics, University of Illinois Urbana-Champaign, Urbana, Illinois, United States of America.*
[2] *Carl R. Woese Institute for Genomic Biology, University of Illinois Urbana-Champaign, Urbana, Illinois, United States of America.*
[3] *Department of Physics, Yale University, New Haven, Connecticut, United States of America.*
[4] *International Centre for Theoretical Sciences, Tata Institute of Fundamental Research, Bengaluru, India.*
[5] *Department of Bioengineering, University of Illinois Urbana-Champaign, Urbana, Illinois, United States of America.*

∗ maslov@illinois.edu; akshitg@icts.res.in

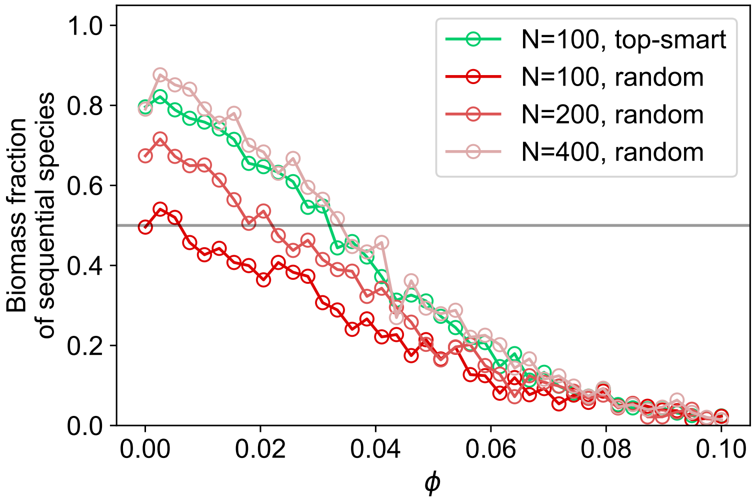

Fig A: **Relaxing the "top-smart" assumption of sequential species preference order.** Green: Same as the green line in Fig 2E, where pool size $N = 100$, and all sequential species are top-smart. Red: red lines of different shades (pool size from 100 to 400, with 1:1 fraction of sequential to co-utilizing strategists) correspond to community assembly outcomes, where sequential species have random preference orders. In these simulations, since the probability of being "top-smart" is $1/n_R = 1/4$, achieving a similar level of sequential species prevalence requires a species pool approximately 4 times larger than in our main simulations (green).

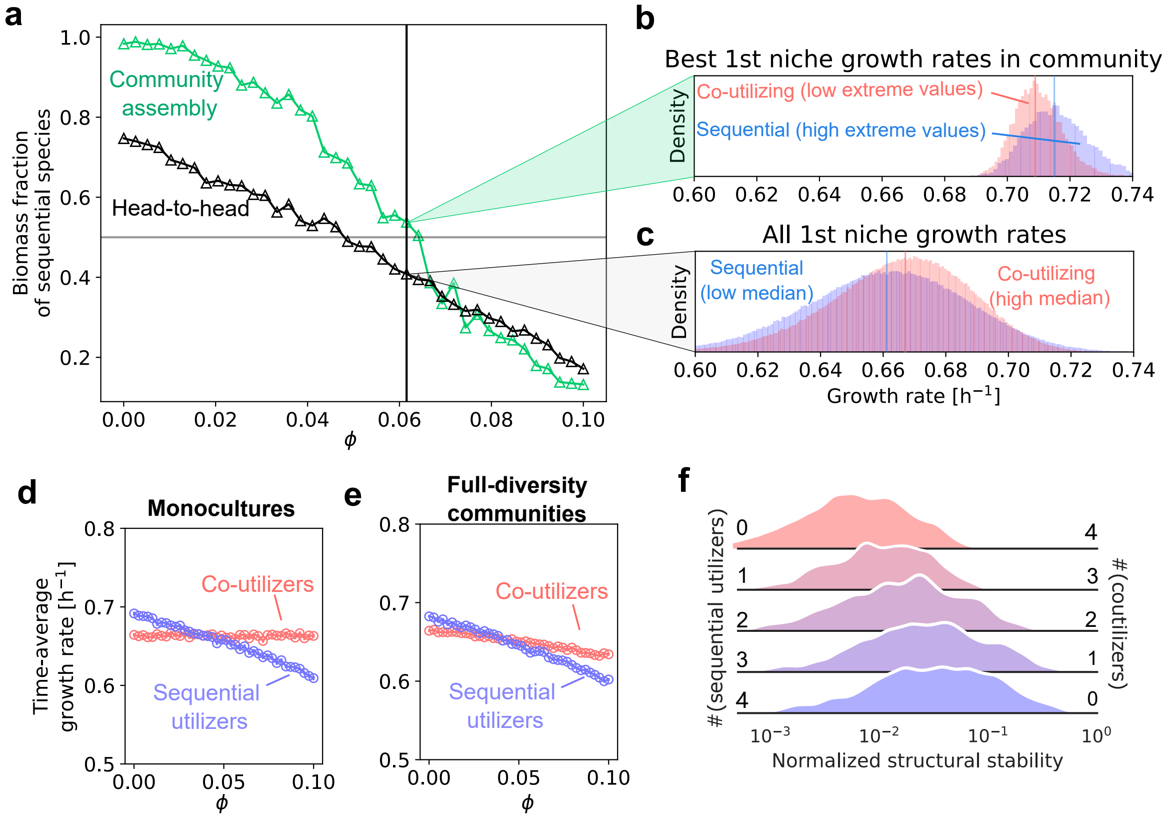

Fig B: **Ecological effects of metabolic strategies when lags are absent.** (a) Fraction of surviving sequential utilizers in each of the two scenarios: head-to-head (black) and in complex communities (green), plotted as a function of the pre-allocation fraction ϕ common to all species in the pool. In the absence of lags, the competitive performance of sequential species is significantly enhanced: compared to the results with lags (Fig 2E), their biomass fraction in head-to-head competitions exhibits as high as $\sim$5-fold increase, while the increase in assembled communities is more moderate. The gray horizontal line denotes competitive parity (biomass fraction=0.5). The black vertical line lies at $\phi = 0.0615$, where sequential species hold a slight advantage in communities, but a slight disadvantage in head-to-head competitions. (b) Distributions of the maximum growth rates sampled from the underlying populations shown in (c); sequential species possess greater extreme values due to their broader distribution tails, enabling them to dominate in complex communities where competition is dictated by extreme value statistics. (c) Population-level growth rate distributions of sequential (blue) and co-utilizing (red) species in the first temporal niche at $\phi = 0.0615$, highlighting that sequential species exhibit higher variance despite a lower mean.(d)-(e) Time-average growth rates of communities as a function of ϕ. (d) In monocultures, the sequential utilizers (blue) gain a growth advantage over coutilizers (red) at low ϕ, which is also reflected in (a). (e) In fully-packed communities, the trend is similar. (f) Distributions of normalized structural stability for niche-packed communities ($n_S = n_R$ species). Apart from the lag time being zero, all other parameters used to generate these communities are identical to the ones in Fig 3C. Increasing the number of sequential utilizers from 0 to 4 systematically increases structural stability.

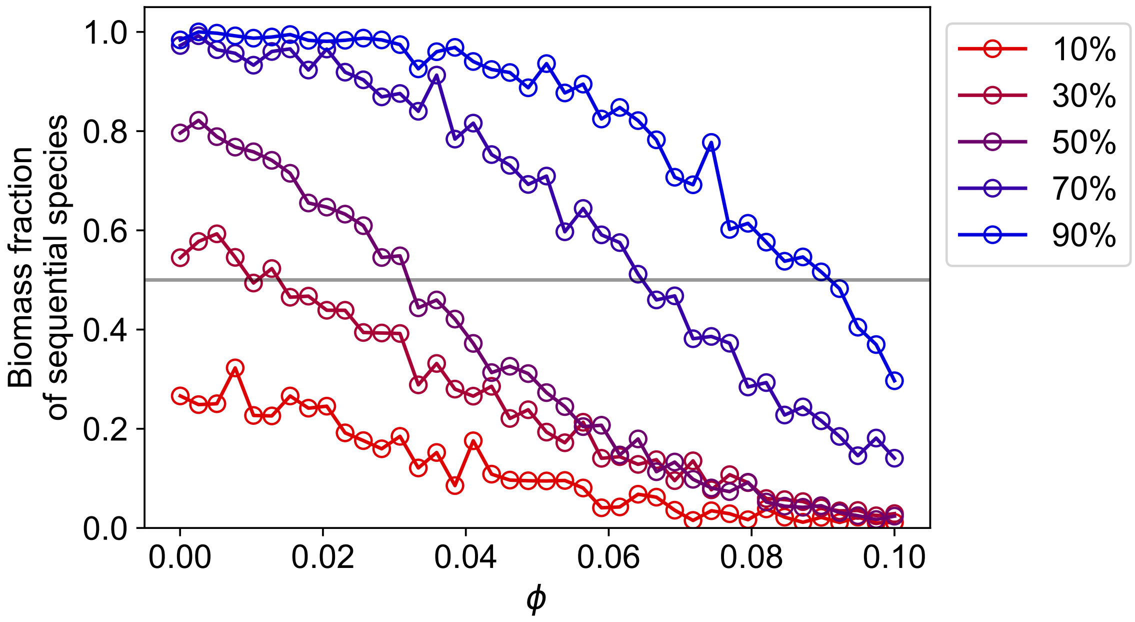

Fig C: **Sensitivity of community composition to the initial composition (sequential species proportion) of the species pool** Final biomass fraction of sequential species across varying initial pool compositions (10%–90% of sequential species). Across the regime of small ϕ ($0 < \phi < 0.05$), sequential species exhibit systematic enrichment, often exceeding their initial pool representation and achieving dominance even when starting as a numerical minority.

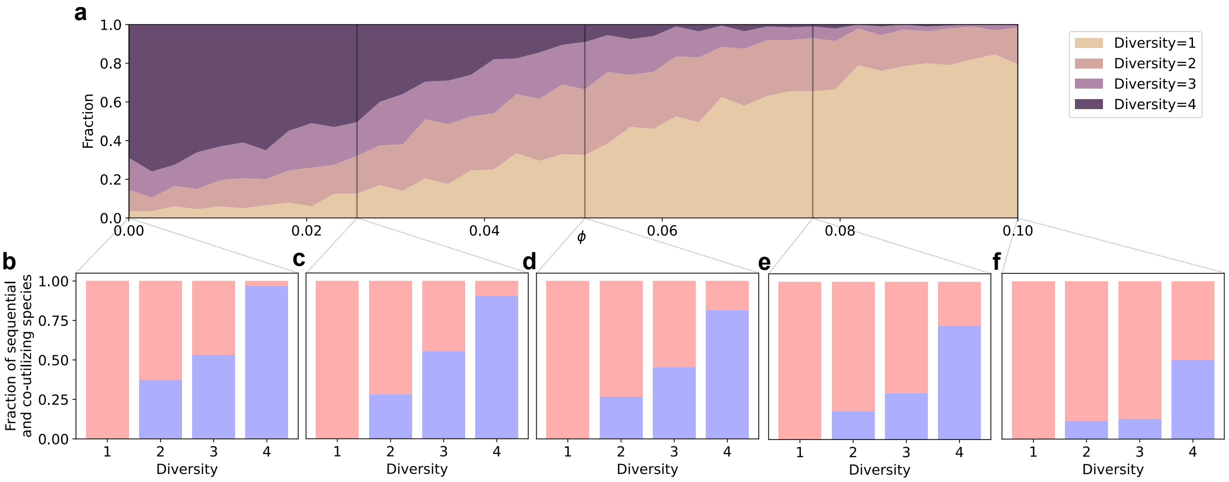

Fig D: **Composition of assembled communities stratified by diversity across different values of ϕ.** (a) Same as Fig 3B. (b)-(f) Stacked bar plot showing how sequential (red) and co-utilizing species (blue) are stratified across communities with different diversities, at specific ϕ values marked on (a).

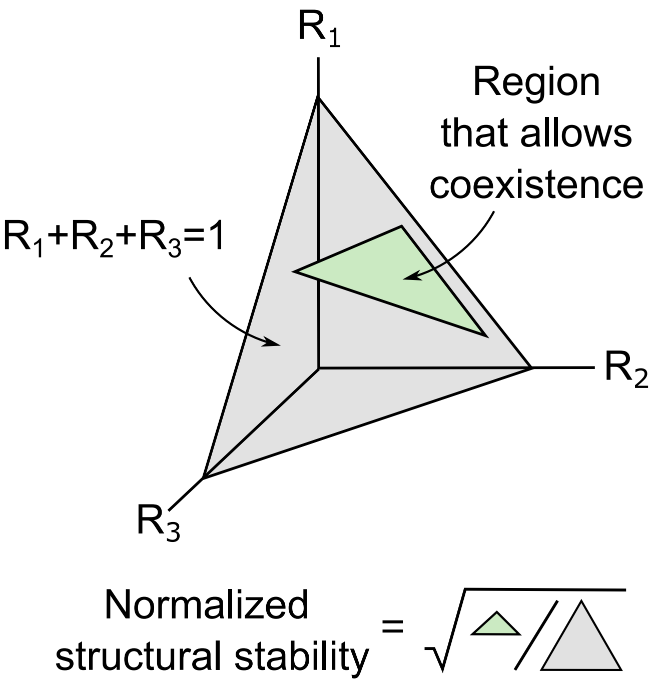

Fig E: **Schematic of structural stability.** Structural stability quantifies the fraction of supplied resource concentration ratios in which a feasible community can assemble (see Methods). It is defined as the fraction of resource supply ratios that support the coexistence of a given microbial community (green) in all possible resource supply ratios (gray).

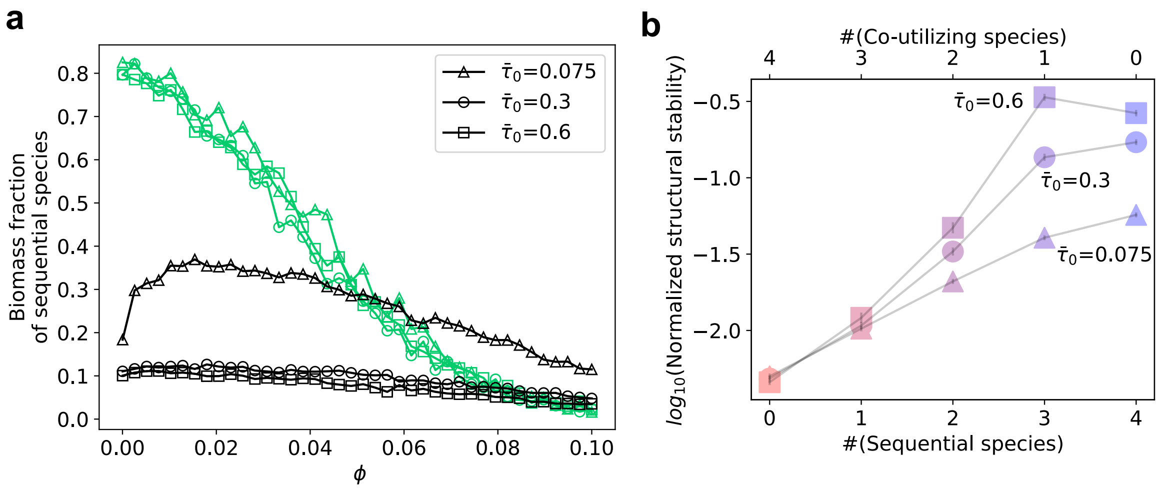

Fig F: **Effects of lags on the ecological advantage of sequential species.** (a) Fraction of sequential utilizers in biomass among survivors with different lags: head-to-head (black) and in complex communities (green), plotted as a function of the pre-allocation parameter ϕ common to all species in the pool. Simulations were performed in the same setup as in Fig 2E. Marker shapes indicate the mean coefficient $\bar{\tau}_0$ of proteome reallocation lag. The sequential lag is calculated by $\tau = \tau_0 \log\left(\frac{1-(n_R-1)\phi}{\phi}\right)$ where the coefficient τ_0 is sampled from a uniform distribution $\bar{\tau}_0 \cdot \mathrm{U}(0.67, 1.33)$ (Methods). (b) The structural stability of niche-packed communities depends on $\bar{\tau}_0$. Simulations were performed in the same way as in Fig 3C, where the average logarithm of normalized structural stability (y-axis) is plotted as a function of the composition of niche-packed communities ($n_S = n_R = 4$). Marker shapes represent the value of $\bar{\tau}_0$, and colors reflect the number of sequential/co-utilizing species. When generating these communities ϕ was taken at 0.036 (Fig 3B).

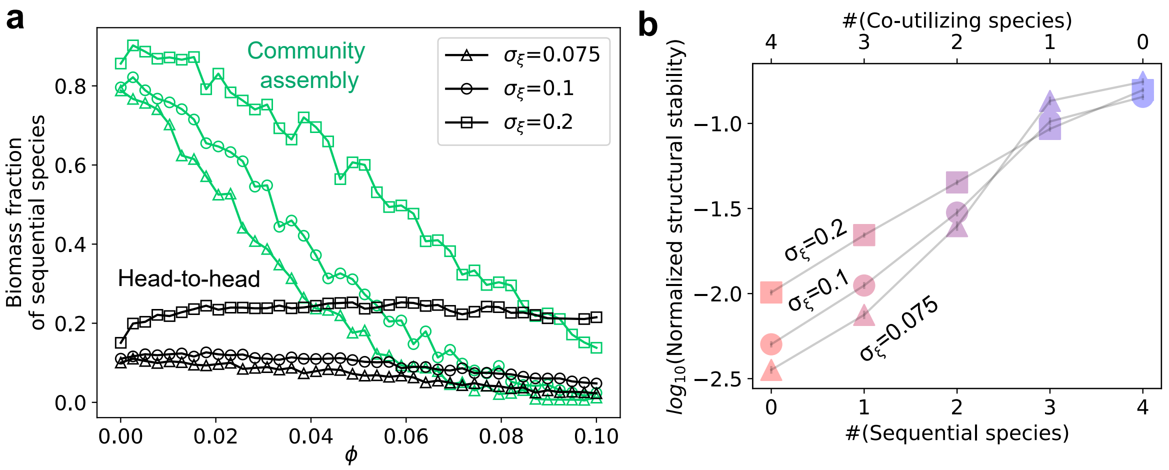

Fig G: **Effects of growth rate distribution on the ecological advantage of sequential species.** (a) Fraction of sequential utilizers in biomass among survivors, in each of the two scenarios: head-to-head (black) and in complex communities (green), plotted as a function of the pre-allocation parameter ϕ common to all species in the pool. Simulations were performed in the same setup as in Fig 2E. Marker shapes indicate the width of enzyme efficiency distribution σ_ξ of $\xi_{\alpha k}$, which contributes to the growth rates. (b) The structural stability of niche-packed communities depends on σ_ξ. Simulations were performed in the same way as in Fig 3C, where the average logarithm of normalized structural stability (y-axis) is plotted as a function of the composition of niche-packed communities ($n_S = n_R = 4$). Marker shapes represent the value of σ_ξ, and colors reflect the number of sequential/co-utilizing species. When generating these communities ϕ was taken at 0.036 (Fig 3B).

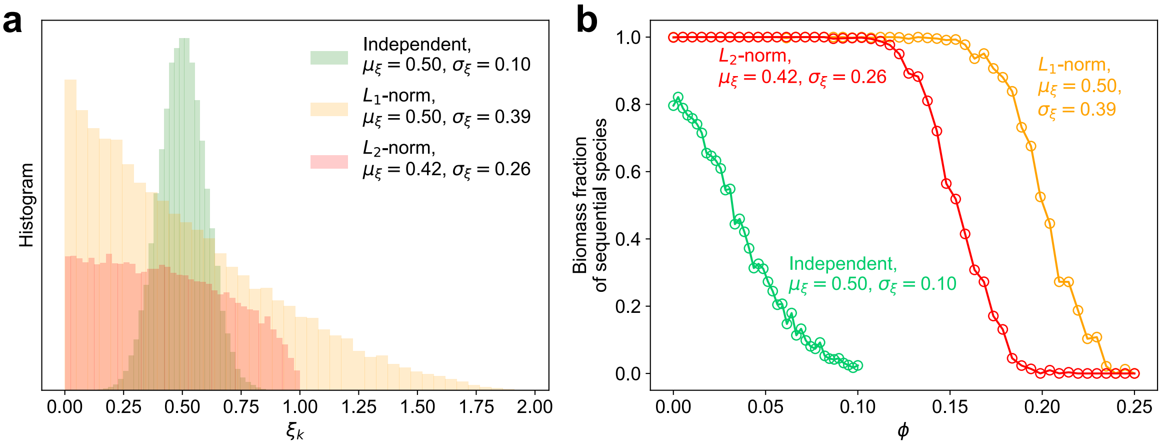

Fig H: **Impact of physiological trade-offs on metabolic strategy fitness (a)** Distribution of enzyme efficiencies ξ_k under different sampling schemes: independent sampling (green), L_1-norm constraint (yellow), and L_2-norm constraint (red). L_p-norm constraints are defined by $\sum_k \xi_{\alpha k}^p = \sum_k \xi_0^p$, where $\xi_0 = 0.5$ as specified in Methods. The introduction of L_p-norm constraints significantly increases the coefficient of variation (σ_ξ/μ_ξ) of efficiencies across resources. The histogram was constructed using $\xi_{\alpha k}$ from 10,000 randomly sampled species for each scheme. **(b)** Biomass fraction of sequential species as a function of the secondary allocation fraction ϕ. Constraints that impose trade-offs between resources (red and orange curves) shift the dominance of sequential species to larger values of ϕ, indicating that physiological trade-offs enhance the ecological advantage of the sequential strategy.

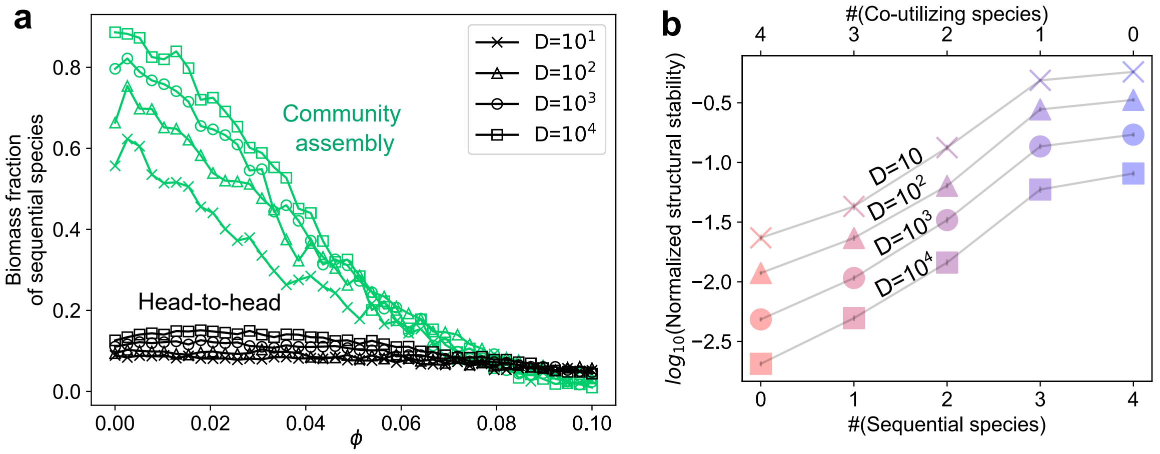

Fig I: **Effect of dilution factor D on the ecological advantage of sequential species.** (a) Fraction of sequential utilizers in biomass among survivors, in each of the two scenarios: head-to-head (black) and in complex communities (green), plotted as a function of the pre-allocation parameter ϕ common to all species in the pool. Simulations were performed in the same setup as in Fig 2E. Marker shapes indicate the dilution factor D. The growth period is shorter under lower D, making the sequential lag's negative effects more pronounced. (b) The structural stability of niche-packed communities depends on D. Simulations were performed in the same way as in Fig 3C, where the average logarithm of normalized structural stability (y-axis) is plotted as a function of the composition of niche-packed communities ($n_S = n_R = 4$). Marker shapes represent the value of D, and colors reflect the number of sequential/co-utilizing species. When generating these communities ϕ was taken at 0.036 (Fig 3B).

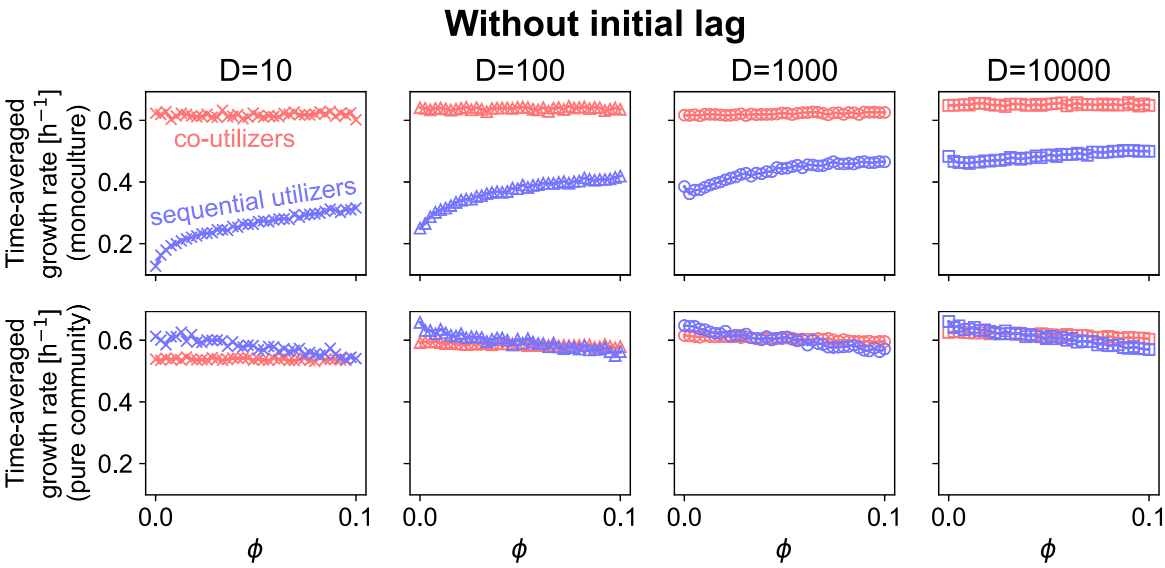

Fig J: **Impact of the dilution factor D on time-averaged growth rates in the absence of initial lag** All simulations shown here were performed by setting the universal initial lag to zero ($\tau_{initial} = 0$). This isolates the specific physiological cost of resource-switching and demonstrates how environmental timescales (governed by D) modulate the inherent fitness differences between metabolic strategies. Monocultures (Top Row): Time-averaged growth rates of co-utilizing (red) and sequential (blue) species as a function of the secondary allocation fraction ϕ for various dilution factors ($D = 10, 100, 1000, 10000$). In monocultures, increasing D lengthens the growth phase while switching lags (τ_{lag}) remain fixed, thereby amortizing the metabolic switching penalty over a longer cycle and improving the relative fitness of sequential utilizers. Full-diversity pure communities (Bottom Row): Time-averaged growth rates in communities comprising four species of the same strategy ($n_S = n_R = 4$). In these niche-packed contexts, sequential species effectively partition resources into a temporal relay, which minimizes realized lags and renders the steady-state growth rate largely insensitive to D compared to the monoculture case.

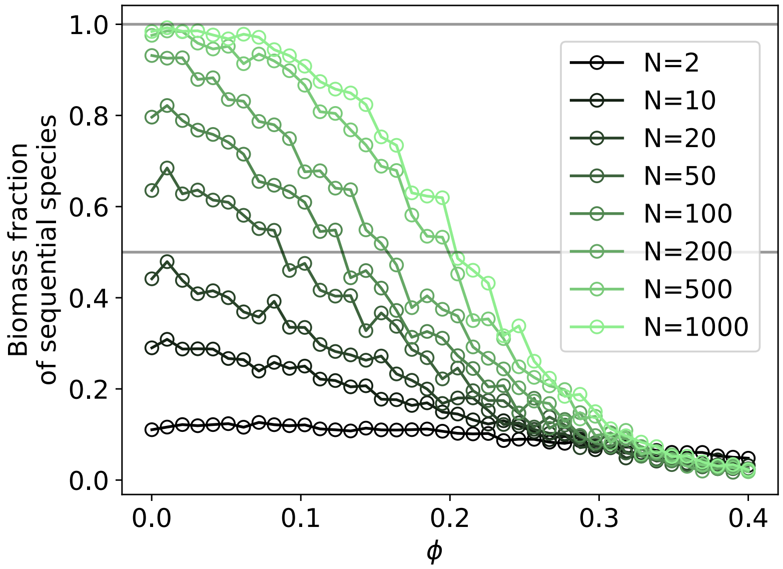

Fig K: **Pool size of assembly affects the ecological advantage of sequential utilizers.** Fraction of sequential utilizers in biomass among survivors (y-axis) are plotted at different pool sizes of the community assembly (represented by different shades of green), as a function of the pre-allocation factor ϕ. For each value of ϕ, we randomly generated 200 species pools, and each of them consists of 50% top smart sequential species and 50% co-utilizing species whose growth rates are sampled from the same distribution as in main text (Fig 2D). The gray horizontal line is at $y = 0.5$.
