# Supplementary material for: Fitness advantage of sequential metabolic strategies emerges from community interactions in strongly fluctuating environments
Source: PLoS Comput Biol. 2026 May 12;22(5):e1014277. doi: 10.1371/journal.pcbi.1014277 (PMC13232955; doi:10.1371/journal.pcbi.1014277)
Supplement: Attachment — Submitted filename: Review_of_Wang_et_al.pdf [file pcbi.1014277.s002.pdf]

Review of Wang et al.,

Fitness Advantage of Sequential Metabolic Strategies Emerges from Community Interactions in Strongly Fluctuating Environments

December 3, 2025

Summary

Wang et al. present a theoretical study of microbial metabolic strategies in boom-and-bust environments, focusing on sequential (diauxic) versus co-utilization of substitutable nutrients. They develop a novel consumer-resource model that integrates dynamic proteome allocation and lag-time penalties from metabolic reconfiguration. Two complementary definitions of strategy fitness are introduced: time-averaged growth rate and biomass-weighted prevalence across community assemblies. Although sequential utilizers are disadvantaged in monoculture competitions due to lag costs, the authors find that they often dominate in diverse, mature communities. This advantage emerges from complementary resource preferences and temporal partitioning, which enhance community coexistence and structural stability. The findings provide insight into the ecological resilience of diauxic strategies and may help explain their prevalence in natural microbial ecosystems.

Overall, in our opinion, this study presents a thought-provoking model and set of predictions about metabolic strategies in microbial communities. However, several issues need clarification, as listed below.

Major Comments

1. A core finding is that while lag time hurts sequential species in isolation, it matters less in communities because species often deplete their top resource and have no resources left to switch to (due to depletion by others). But if a species stops growing because it has no resources left to switch to, its total biomass should presumably be low. Readers may be confused as to why "having nothing left to eat, thus avoiding the lag penalty" translates into a success strategy (biomass prevalence). The authors need to clarify how this limitation actually results in a competitive edge.
2. In our opinion, the secondary allocation scheme is reasonable but idealized. It abstracts away any resource-specific differences in enzyme costs or kinetics. For instance, by construction, it assumes co-utilizers allocate evenly across resources, whereas sequentials invest fully in the highest-priority resource (i.e., $|S_{\text{primary}}| = 1$). This dichotomy (a single primary vs. many) may exaggerate the sequential strategy's ability to focus its proteome. In reality, microbes may face trade-offs between enzyme efficiencies $\xi_{\alpha k}$ and adjustments to proteome fractions. Notably, the authors assume that each species' growth rate on different single resources is chosen independently (i.e., no physiological trade-offs). While this maximizes diversity, it might not hold for real microbes, where specialization on one carbon source often reduces performance on another. If such trade-offs exist, they could

mitigate the advantage of breadth (co-utilization) or narrowness (sequential) in unforeseen ways. It would strengthen the manuscript to discuss how relaxing the independence assumption might change the results, or at least to cite related work on trade-offs (e.g. expected dilution experiments).

3. The paper introduces two notions of fitness: 1. Time-averaged growth rate, and 2. Biomass-weighted prevalence. In the results, the authors switch between these metrics. However, at steady state, all surviving species must have the same time-averaged growth rate (equal to the dilution factor D). The text explains they used "pure" communities to overcome this comparison difficulty, but this logical pivot can be disorienting. Readers might get lost regarding which metric is the true standard for "winning."
4. The model assumes all sequential species are "Top-Smart," meaning they always utilize the resource that provides the fastest growth rate first. But in nature, microbes do not always know which resource is "best" (e.g., suboptimal diauxie). Readers may question if the conclusion holds if sequential utilizers choose the "wrong" order, or if this strong assumption artificially inflates the advantage of the sequential strategy.
5. After growth to the stationary phase, biomass is diluted by a factor D and fresh resources are supplied. The dilution factor is a crucial parameter: it sets the severity of the bust phase. The authors set $D = 1000$ (24 hr cycles) for most experiments — an very strong die-off each cycle — arguably more extreme than typical laboratory serial-dilution experiments. The authors briefly note (SM) that varying D has only a small effect on the relative fitness of strategies. While this suggests robustness, it would be useful to see more intuition or references: e.g. how does the time-averaged growth metric (Fig. 2a) depend on D? They note in passing (SM) that larger D yields stronger busts but only minor changes in sequential advantage. A further parameter sweep or even a qualitative discussion of D would help.
6. The manuscript provides only a brief, intuitive definition of structural stability (the range of supply conditions). It would be valuable to connect this to established theory; for example, studies by Rohr et al. and others on the feasibility domains of coexistence. Why should sequential specialists have greater structural stability? The authors suggest that it (implicitly) might be due to the variance in growth-rate distributions (Suppl. Fig. S3) – sequential species have greater growth-rate variance because they do not average across resources. A clearer mechanistic discussion would help. Perhaps they could analytically or qualitatively argue how sequential consumption creates nested (temporal) niches that buffer against fluctuation, whereas co-utilizers require simultaneous resource supply.
7. The simulations use $n_R = 4$ total resources and sample communities up to 4 species surviving. How well do the results generalize to more complex systems? - A comment on this would be good.
8. The authors focus exclusively on serial dilution dynamics and do not explore extensions to continuous or spatial environments, cross-feeding networks, or adaptive enzyme strategies. Serial dilution is a particular scenario; more discussion on the possibility of such temporal niche segregation in continuous or spatial environments is warranted. For example, while they say, "While our results are based on a serial dilution protocol, we expect similar dynamics to arise in environments that exhibit repeated depletion and replenishment of resources...", they stop short of providing supporting evidence. Also, what about the robustness of the results to explicit metabolite production and uptake (cross-feeding networks)? For example, would sequential dominance in high-diversity assemblies be further amplified or counterbalanced by facilitative cross-feeding loops, provided the regime is still serial dilution?

Minor Comments and Suggestions

- In the Abstract and later, the word "mature" is undefined.
- In the Methods, Line 337, the text presents formulas with logs and fractions of ϕ and n_R without a physical/biological explanation. Non-math readers will not intuitively grasp why increased diversity (n_R) or allocation (ϕ) affects the lag time τ just by looking at the equation. We recommend adding some text descriptions.
- The model also includes ribosomal–metabolic proteome sector dynamics (as in Scott et al. 2010 [cited by Wang et al]) such that both sectors change with growth rate. However, details of these dynamics are briefly mentioned but not sufficiently described in the main text. It might help readers to see, for example, a statement of how $E_\alpha^{(m)}$ is updated each cycle, or a reference to the specific supporting equation. The Methods section mentions a standard linear allocation rule based on the Scott et al. model, but a summary in the main text could improve clarity.
- The term "structural stability" is introduced without citation (e.g. cite Pettersson & Case or Rohr et al.).
- In Fig. 2d-e, "pure" communities are constructed with equal numbers (50 each) of sequential and co-utilizer species. The authors should justify this choice. How sensitive are results to the relative pool sizes? If the pool were biased (say 70 sequentials, 30 co-utilizers), would sequentials always dominate? Also, the study focuses on two extreme cases (one-on-one vs 100-species mixed). It might strengthen the argument to show intermediate scenarios (e.g. small consortia of 5–10 species) to show if/how the advantage scales with community size.
- Mathematically define "time-averaged growth rate" at first use in main text (e.g. $\langle g \rangle$ or $\Delta \ln(\text{biomass})/\Delta t$).

Samraat Pawar and Yan Zhu,
Silwood Park, Imperial College London
