# Supplementary material for: Fitness advantage of sequential metabolic strategies emerges from community interactions in strongly fluctuating environments
Source: PLoS Comput Biol. 2026 May 12;22(5):e1014277. doi: 10.1371/journal.pcbi.1014277 (PMC13232955; doi:10.1371/journal.pcbi.1014277)
Supplement: Attachment — Submitted filename: niche_complementarity_plos_reviewer_responses.pdf [file pcbi.1014277.s004.pdf]

Review file for *Fitness Advantage of Sequential Metabolic Strategies Emerges from Community Interactions in Strongly Fluctuating Environments*

Reviewer 1

In this manuscript, Wang et al. study the competition between microbial species with different metabolic strategies in ecological communities under boom-and-bust cycles. For this, the authors develop a Consumer-Resource model that captures the cyclic arrival of nutrients into the system in a way analogous to serial dilutions experiments. Co-utilizing species divide their enzyme budget—and therefore average their growth rate— among the available (or primary) resources, and sequential utilizers use resources one at a time, which can maximize growth rate on their preferred resource at a cost of a potential lag time when switching to another resource.

The paper was a pleasure to read, I found it insightful and well written. I especially appreciate the clear explanation on how smaller enzyme budget fractions allocated to every other primary resource favors sequential consumers in community context, the linear algebra approximation providing an interpretable prediction on the structural stability of niche-packed communities, and the experimentally testable predictions on the prevalence of sequential consumers in high diversity communities—left as a proposal for experimentalists to test in the future. I recommend this manuscript for publication in PLOS Computational Biology, with just some minor potential changes. Below are some suggestions and small comments that I have.

We thank the reviewer for the encouraging remarks and for finding our study interesting. We are grateful for the constructive suggestions provided, which have helped us further clarify our model and strengthen the manuscript.

I think that the second '=' sign in Eq. 1 is a typo. I would expect it to be replaced by a product sign '·', or another functional form. Otherwise, I don't understand how both E^r =1 and E^m =1 maximize the growth rate, and an explanation should be given.

We thank the reviewer for this comment and for pointing out the potential confusion caused by the notation in Eq. 1.

The reviewer is correct that the second '=' sign does not represent a definition, but rather a flux-matching condition, characteristic of proteome allocation models at steady state (Scott et al., Science, 2010). The growth in our model is limited by 2 processes: the translation capacity $g_C E_\alpha^{(r)}$ which is proportional to the fraction of proteins that are ribosomes, and the nutrient uptake capacity $(\sum \xi_{\alpha k} x_{\alpha k}) E_\alpha^{(m)}$, which is proportional to the fraction of proteins that are metabolic enzymes. At the optimal or steady-state growth rate, these two fluxes must be balanced or equal, so that neither precursors nor ribosomes accumulate unnecessarily. This

balance, together with the allocation constraint that these fractions sum to a constant $E_{\alpha}^{(r)} + E_{\alpha}^{(m)} = 1$ allows us to derive the overall growth rate g_{α}.

We agree that the previous notation was somewhat ambiguous. We have now revised the text (line 338-344) to explicitly state that the growth rate is determined by the balance of these two fluxes and have rewritten Eq. 1 to make this derivation more transparent.

The model assumes top-smart sequential species. How would the relaxation of this assumption affect the results? Perhaps it would be interesting to compare with a top-random choice (or any other illustrative choice). Intuitively, this would make it more difficult to find competitive-enough sequential species, increasing the diversity of the species pool required to generate niche-packed communities. But I'd be interested in reading the interpretation—and potentially some analysis— of the authors.

We thank the reviewer for this insightful comment. The reviewer's intuition is correct: relaxing the "top-smart" assumption makes it significantly harder for sequential species to remain competitive. In our model, competition is most intense during the first temporal niche when all resources are available. A sequential species' growth rate in this critical niche is determined solely by its most-preferred resource. Therefore, a sequential species that is not "top-smart" is highly susceptible to being outcompeted by co-utilizers or other top-smart sequential species.

We have revised the text (line 119-125) to clarify that the "top-smart" strategy is not merely an assumption of optimality, but rather the dominant sequential strategy that emerges from community assembly during serial dilutions, as we showed in our previous work (Wang et al., Nature Communications, 2021, Fig. 2d).

To further address this concern, we also performed additional simulations where sequential species are assigned random preference orders, rather than being "top-smart" to begin with. We still observe that sequential species both prevail and are dominant, but with a larger species pool, approximately n_R times larger than in the main text ($n_R = 4$ is the number of resources provided). This is expected, since it is still the top-smart species that are being selected for by the community assembly dynamics. It is just that the probability of being top-smart is now $1/n_R$ instead of 1 (as in the main text). We have now included these results in the Supplementary Information (Fig. S1).

Check the format of all references. For example, there's a problem with the citation in line [291].

Another typo, this one about Sr in line 372

Line 107, typo '…it takes TO deplete all…'. Check the text for other minor typos like this, e.g. line 122.

We thank the reviewer for identifying these errors. We have performed a comprehensive revision of the text to fix them, as well as further ensure technical and grammatical accuracy.

In the caption of Fig. S5, some quantity is said to 'increase by a lot', which was a bit funny to read (please replace by a more quantitative or rigorous vocabulary). Also in the same caption, 'y=5' but 'y' has not been defined as biomass fraction. Same caption, the description of panel c precedes the one of b.

We thank the reviewer for pointing out these issues. We agree that the previous wording was informal and lacked precision, and we have revised the caption of Fig. S5 (now Fig. S2).

I think that the supplementary figures should be indexed according to their order of citation in the main text. Fig. S5 is the first one to be cited. I think most of the other supplementary figures are cited later in the main text, but I did not check if all of them are cited.

We agree with the reviewer's suggestion. We have re-indexed all supplementary figures to ensure they appear in the order in which they are first cited in the main text. We have also verified that every supplementary figure is explicitly cited and correctly referenced within the manuscript.

Thanks again for this reading, I found it very interesting.

We are grateful for the reviewer's encouraging comments and for the interest shown in our work. We thank the reviewer again for the thoughtful and constructive suggestions, which have been very helpful in improving our manuscript.

Reviewer 2

Wang et al. present a theoretical study of microbial metabolic strategies in boom-and-bust environments, focusing on sequential (diauxic) versus co-utilization of substitutable nutrients. They develop a novel consumer-resource model that integrates dynamic proteome allocation and lag-time penalties from metabolic reconfiguration. Two complementary definitions of strategy fitness are introduced: time-averaged growth rate and biomass-weighted prevalence across community assemblies. Although sequential utilizers are disadvantaged in monoculture competitions due to lag costs, the authors find that they often dominate in diverse, mature communities. This advantage emerges from complementary resource preferences and temporal partitioning, which enhance community coexistence and structural stability. The findings provide insight into the ecological resilience of diauxic strategies and may help explain their prevalence in natural microbial ecosystems.

Overall, in our opinion, this study presents a thought-provoking model and set of predictions about metabolic strategies in microbial communities. However, several issues need clarification, as listed below.

We thank the reviewer for their rigorous reading and insightful suggestions, which we think greatly improved our manuscript.

Major Comments

1. A core finding is that while lag time hurts sequential species in isolation, it matters less in communities because species often deplete their top resource and have no resources left to switch to (due to depletion by others). But if a species stops growing because it has no resources left to switch to, its total biomass should presumably be low. Readers may be confused as to why "having nothing left to eat, thus avoiding the lag penalty" translates into a success strategy (biomass prevalence). The authors need to clarify how this limitation actually results in a competitive edge.

We thank the reviewer for highlighting this potential point of confusion.

While it is true that each species in a community of sequential utilizers achieves lower biomass than they would in isolation, in boom-and-bust environments it is not high biomass but short depletion times that are selected for. Thus the "competitive edge" of a species comes due to its large growth rate on its top choice, rather than its biomass. We thank the reviewer for pointing out this potential source of confusion. We have now clarified this in the revised text (lines 148-149).

2. In our opinion, the secondary allocation scheme is reasonable but idealized. It abstracts away any resource-specific differences in enzyme costs or kinetics. For instance, by construction, it assumes co-utilizers allocate evenly across resources, whereas sequentials invest fully in the highest-priority resource (i.e., $|S_{primary}| = 1$). This dichotomy (a single primary vs. many) may exaggerate the sequential strategy's ability to focus its proteome. In reality, microbes may face trade-offs between enzyme efficiencies $\xi_{\alpha k}$ and adjustments to proteome fractions. Notably, the authors assume that each species' growth rate on different single resources is chosen independently (i.e., no physiological trade-offs). While this maximizes diversity, it might not hold for real microbes, where specialization on one carbon source often reduces performance on another. If such trade-offs exist, they could mitigate the advantage of breadth (co-utilization) or narrowness (sequential) in unforeseen ways. It would strengthen the manuscript to discuss how relaxing the independence assumption might change the results, or at least to cite related work on trade-offs (e.g. expected dilution experiments).

We thank the Reviewer for this thoughtful comment regarding the assumption of independence in enzyme efficiencies ($\xi_{\alpha k}$).

We would like to first clarify that our model does explicitly incorporate a fundamental trade-off through the proteome allocation budget ($x_{\alpha k}$): allocating more enzymes to one resource necessarily reduces the investment in others.

To address the reviewer's concern about the lack of resource-specific trade-offs in enzyme efficiencies ($\xi_{\alpha k}$), we additionally performed simulations, where we imposed physiological

constraints on the kinetic parameters $\xi_{\alpha k}$, such as the L_p-norm constraints ($\sum_k \xi_{\alpha k}{}^p = const.$), where we used p=1 and 2. Our results, now detailed in Fig. S8, show that our conclusions are not only robust to these trade-offs but that such constraints actually enhance the advantage of sequential strategies. Mechanistically, imposing a trade-off increases the variance (standard deviation) of the $\xi_{\alpha k}$ distribution across resources compared to independent sampling. Since sequential utilizers rely on their maximum efficiency ($max_k(\xi_{\alpha k})$) while co-utilizers rely on the average efficiency ($mean_k(\xi_{\alpha k})$), this increased variance raises the $max_k(\xi_{\alpha k})/mean_k(\xi_{\alpha k})$ ratio, providing an even greater competitive edge to diauxic species (Methods Eq. 3). We have now addressed this in the Discussions (line 249-255).

3. The paper introduces two notions of fitness: 1. Time-averaged growth rate, and 2. Biomass-weighted prevalence. In the results, the authors switch between these metrics. However, at steady state, all surviving species must have the same time-averaged growth rate (equal to the dilution factor D). The text explains they used "pure" communities to overcome this comparison difficulty, but this logical pivot can be disorienting. Readers might get lost regarding which metric is the true standard for "winning."

We thank the Reviewer for highlighting this potential point of confusion. We have clarified the relationship between these two metrics and the logical role of "pure" communities in our revised manuscript (line 135-136; 151-152; 157-158).

First, we wish to clarify a technical detail regarding the time-averaged growth rate: even though all coexisting species have the same time-averaged growth rate at steady state, this rate is not the dilution factor *D*, but $\log D/T_{dep}$, where T_{dep} is the time required to deplete all resources. Although D is a constant, T_{dep} is a community-level property that varies depending on the strategies present. Therefore the time-averaged growth rate can change from community to community, rather than being a constant equal to *D*.

Regarding the narrative flow, we agree that biomass-weighted prevalence is the ultimate standard for "winning" in an ecological competition. We used the time-averaged growth rate primarily as a mechanistic tool to build intuition. Our logical pivot follows the following progression:

- Monoculture: We use the growth rate to show that sequential species are inherently disadvantaged due to lag times.
- Pure Communities (Controlled Experiment): Since mixed-strategy communities constrain all species to the same growth rate, we introduced "pure" communities as a controlled comparison. This isolates how the community context (specifically complementarity) neutralizes the individual lag penalty.

- Mixed Pools: Finally, we confirm that this mechanistic recovery of sequential species indeed translates into ecological success using the definitive standard of biomass prevalence in competitive assembly.

We have updated the text to guide the reader through this transition more clearly.

4. The model assumes all sequential species are "Top-Smart," meaning they always utilize the resource that provides the fastest growth rate first. But in nature, microbes do not always know which resource is "best" (e.g., suboptimal diauxie). Readers may question if the conclusion holds if sequential utilizers choose the "wrong" order, or if this strong assumption artificially inflates the advantage of the sequential strategy.

We thank the reviewer for this insightful comment. We agree that it is important to verify whether the "top-smart" assumption influences the robustness of our conclusions or artificially inflates the success of sequential strategies. To address this, we have added a new sensitivity analysis in Fig. S1, where we relax this assumption by assigning random resource preference orders to sequential species.

We find that the ecological advantage of the sequential strategy is qualitatively robust to this assumption. The choice of the "top-smart" strategy in our main model is based on its dominance during community assembly, as we showed in previous work (Wang et al., Nature Communications, 2021, Fig. 2d). This occurs because competition is most intense during the first temporal niche when all resources are available. Any species that grows relatively slower during this period—by most preferring a suboptimal resource on which it grows slower—is very likely going to get outcompeted by either co-utilizers or "top-smart" specialists.

The new simulations confirm that sequential species continue to be dominant in a species pool that is sufficiently large. The need for a larger pool follows from the increased number of combinatorial possibilities for different sequential strategies now that they are not necessarily top-smart. Specifically, because the probability of a random species being top-smart is now $1/n_R$ (where $n_R = 4$ is the number of resources provided), a species pool approximately n_R times larger is required to match the typical number of top-smart sequential species compared to the previous pool. This is exactly when our results start to match with the main text. Taken together, these results further support our claim that the success of sequential utilization is a robust feature of the community dynamics rather than an artifact of a specific preference order. We have updated the manuscript (lines 119-125) to clarify this point.

5. After growth to the stationary phase, biomass is diluted by a factor D and fresh resources are supplied. The dilution factor is a crucial parameter: it sets the severity of the bust phase. The authors set D=1000 (24 hr cycles) for most experiments an very strong die-off each cycle arguably more extreme than typical laboratory serial-dilution experiments. The authors briefly note (SM) that varying D has only a small effect on the relative fitness of strategies. While this suggests robustness, it would be useful to see more intuition or references: e.g. how does the time-averaged growth metric (Fig. 2a) depend on D? They note in passing (SM) that larger D yields stronger busts but only

minor changes in sequential advantage. A further parameter sweep or even a qualitative discussion of D would help.

We thank the reviewer for this opportunity to clarify the range of values we chose for the dilution factor *D* and its mechanistic impact on fitness.

First, we clarify that a dilution factor of *D* ~ 1000 is consistent with recent microbial ecology experiments. For example, Friedman et al. (Nature Ecol. Evol., 2017) utilized a dilution factor of *D* = 1500 in their community assembly experiments. To ensure our results cover the full range of experimentally relevant conditions, we have modified Fig. S9 by also including results for *D*=10. These additional analyses confirm that our qualitative conclusions still hold.

We have revised the Discussion to clarify the relationship between fixed physiological lag time and the environmentally-driven growth cycle (line 259-264). The intuition is that the metabolic lag (τ_{lag}) associated with switching is a fixed physiological constant. As the dilution factor *D* increases, the growth phase ($t_{growth} \propto \log D$) lengthens, meaning this fixed lag time occupies a progressively smaller fraction of the total cycle. Consequently, a higher *D* reduces the relative fitness penalty of the sequential strategy compared to co-utilizers. We have added Fig. S10 to explicitly show how time-averaged growth rates (excluding initial lag) depend on D. In niche-packed communities, species minimize these lags through efficient resource partitioning, rendering the community state largely independent of D.

6. The manuscript provides only a brief, intuitive definition of structural stability (the range of supply conditions). It would be valuable to connect this to established theory; for example, studies by Rohr et al. and others on the feasibility domains of coexistence. Why should sequential specialists have greater structural stability? The authors suggest that it (implicitly) might be due to the variance in growth-rate distributions (Suppl. Fig. S3) sequential species have greater growth-rate variance because they do not average across resources. A clearer mechanistic discussion would help. Perhaps they could analytically or qualitatively argue how sequential consumption creates nested (temporal) niches that buffer against fluctuation, whereas co-utilizers require simultaneous resource supply.

We thank the reviewer for this constructive feedback. We agree that grounding our results more explicitly in a theoretical framework strengthens the manuscript. In response, we have revised the Discussion (line 272-273) and Results (line 189-197) sections to provide a clearer foundation for our findings, and to integrate the frameworks of Rohr et al. (2014) and Butler &

O'Dwyer (2018).

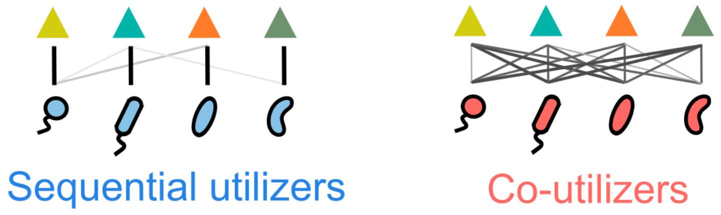

In the framework established by Rohr et al., structural stability is defined as the volume of the feasibility domain, which is increased by decreasing the overall "effective competition" (or niche overlap) between species. In our model, unlike co-utilizers that compete for all resources simultaneously, sequential species partition resources into complementary temporal niches, effectively decreasing their niche overlap over time (see schematic above, where triangles represent resources, and thickness of links represent biomass gained from the resource during the steady-state dilution cycle). This effective niche segregation over time reduces competition between complementary sequential species and expands the feasibility domain of communities comprising them. In this sense, it becomes easier to see why changing the supply concentration of any one resource will simultaneously affect all co-utilizers in the community on the right of the schematic, only but only one of the sequential species in the community on the left. This is why pure sequential communities are much more structurally stable, in the same sense as the frameworks mentioned by the reviewer.

7. The simulations use $n_R = 4$ total resources and sample communities up to 4 species surviving. How well do the results generalize to more complex systems? A comment on this would be good.

We thank the reviewer for this question regarding the generalizability of our findings to more complex settings. We have added a discussion on the robustness of our results to increasing the number of resources n_R. We find that the fitness advantage of sequential strategies is robust to increasing n_R. However, as n_R increases, we need a larger species pool to ensure that the community assembly process has all the complementary sequential species. This is natural and expected because we generate our species pools randomly. Moreover, assembling from a larger species significantly slows down simulations. This is why we chose a manageable number of resources n_R= 4. We have added a comment on this scaling in the Discussion (line 280-288).

8. The authors focus exclusively on serial dilution dynamics and do not explore extensions to continuous or spatial environments, cross-feeding networks, or adaptive enzyme strategies. Serial dilution is a particular scenario; more discussion on the possibility of such temporal niche segregation in continuous or spatial environments is warranted. For example, while they say, "While our results are based on a serial dilution protocol, we expect similar dynamics to arise in environments that exhibit repeated depletion and replenishment of resources...", they stop short of providing supporting evidence. Also,

what about the robustness of the results to explicit metabolite production and uptake (cross-feeding networks)? For example, would sequential dominance in high-diversity assemblies be further amplified or counterbalanced by facilitative cross-feeding loops, provided the regime is still serial dilution?

We appreciate the reviewer's comments regarding the scope and generalizability of our model.

Regarding the specific quote mentioned, we were unable to locate the exact phrase in our current manuscript. However, we fully agree with the underlying point: the environmental context (fluctuating vs. steady-state) is critical for the relevance of sequential metabolic strategies. We have updated the Introduction (line 44-46) to explicitly clarify that steady-state systems like chemostats are fundamentally incompatible with the mechanism of temporal niche segregation. Since resources in such systems do not undergo repeated depletion and replenishment, the ecological conditions required for sequential metabolic strategies to manifest are absent.

As for spatial environments, while we acknowledge that spatial structure adds another layer of complexity, modeling spatial consumer-resource dynamics with such large species pools requires a whole new modeling framework, and in our understanding is beyond the scope of this work. We have added a brief acknowledgement of this as a potential future research direction in the Discussion (line 289-299).

Regarding cross-feeding, we have expanded our discussion on how such interactions might affect sequential dominance. As noted in the last paragraph of Discussion, cross-feeding typically creates "secondary" resources (in the form of metabolic byproducts) that appear later in the growth cycle. In our framework, this effectively potentially changes both the number and sequence of temporal niches. We also argue that we believe such cross-feeding would likely amplify the advantage of sequential utilizers we observed here. This is because sequential species are naturally better equipped to temporally partition these windows of growth without the high cost to simultaneously express several different enzymes paid by co-utilizers.

Minor Comments and Suggestions

- In the Abstract and later, the word "mature" is undefined.

We agree that a clear definition for the term "mature." is needed. We have added a clarifying definition in the Abstract (line 15) and have further elaborated on this in the Introduction (line 52-56) to ensure that "maturity" is explicitly linked to the cumulative number of species invasion attempts (species pool size of community assembly in simulations).

- In the Methods, Line 337, the text presents formulas with logs and fractions of ϕ and n_R without a physical/biological explanation. Non-math readers will not intuitively grasp why increased diversity (n_R) or allocation (ϕ) affects the lag time τ just by looking at the equation. We recommend adding some text descriptions.

We thank the reviewer for this suggestion. We have revised the Methods section (line 387) to provide a clearer physiological intuition behind our lag time equation.

- The model also includes ribosomal–metabolic proteome sector dynamics (as in Scott et al. 2010 [cited by Wang et al]) such that both sectors change with growth rate. However, details of these dynamics are briefly mentioned but not sufficiently described in the main text. It might help readers to see, for example, a statement of how $E_{\alpha}^{(m)}$ is updated each cycle, or a reference to the specific supporting equation. The Methods section mentions a standard linear allocation rule based on the Scott et al. model, but a summary in the main text could improve clarity.

We thank the reviewer for this suggestion. We agree that a brief summary of the sector reallocation rule in the main text would improve clarity. We have added a description in the Results section (line 100-105) stating that the sizes of the metabolic and ribosomal sectors are not fixed but instead adapt dynamically to the instantaneous growth rate.

- The term “structural stability” is introduced without citation (e.g. cite Pettersson & Case or Rohr et al.).

We thank the reviewer for highlighting the importance of properly situating "structural stability" within the literature. We would like to clarify that the citation for Rohr et al. was included in the original manuscript at the first formal definition of the term in the Introduction (line 55 of original manuscript).

- In Fig. 2d-e, “pure” communities are constructed with equal numbers (50 each) of sequential and co-utilizer species. The authors should justify this choice. How sensitive are results to the relative pool sizes? If the pool were biased (say 70 sequentials, 30 coutilizers), would sequentials always dominate? Also, the study focuses on two extreme cases (one-on-one vs 100-species mixed). It might strengthen the argument to show intermediate scenarios (e.g. small consortia of 5–10 species) to show if/how the advantage scales with community size.

We thank the reviewer for highlighting these points. While the scaling of sequential dominance with community complexity was present in our original analysis (Fig. S1), we agree that this relationship deserves more explicit emphasis to ensure it is clear to all readers.

We have revised Results (line 165-168) and included Fig. S3 to demonstrate that sequential species are systematically enriched compared to their initial fraction in the species pool when ϕ is small. We have also revised Testable Predictions (line 312-313) sections to more prominently describe how the sequential advantage scales from small consortia to high-diversity assemblies.

- Mathematically define “time-averaged growth rate” at first use in main text (e.g. $\langle g \rangle$ or $\Delta \ln(\text{biomass})/\Delta t$)

We agree that a formal mathematical definition improves clarity. We have updated the text to define the steady-state time-averaged growth rate as $\log\frac{N(T_{dep})}{N(0)}/T_{dep}$, where T_{dep} is the time it takes to deplete all resources in a single cycle, and N(t) is the biomass of a species at time t (line 114, 116).

Samraat Pawar and Yan Zhu, Silwood Park, Imperial College London
