# Supplementary material for: Fitness advantage of sequential metabolic strategies emerges from community interactions in strongly fluctuating environments
Source: PLoS Comput Biol. 2026 May 12;22(5):e1014277. doi: 10.1371/journal.pcbi.1014277 (PMC13232955; doi:10.1371/journal.pcbi.1014277)
Supplement: Attachment — Submitted filename: review_response_zihan_plos_cb_round_2.pdf [file pcbi.1014277.s005.pdf]

Response to Reviewer Comments on *Fitness Advantage of Sequential Metabolic Strategies Emerges from Community Interactions in Strongly Fluctuating Environments* (2nd Round)

Reviewer 1

In my opinion, the authors have appropriately addressed all the questions and suggestions from the referees. I recommend the current version for publication.

We are grateful to the reviewer for the positive assessment and for recommending our manuscript for publication.

Reviewer 2

The revised manuscript presents a clear and conceptually strong contribution, extending consumer–resource models to incorporate dynamic proteome allocation and demonstrating an emergent ecological advantage of sequential metabolic strategies in diverse communities. The authors have addressed the major concerns from the first round effectively, including robustness to key assumptions (e.g. “top-smart” strategies, parameter trade-offs, dilution factor) and improved clarity around the fitness metrics and mechanisms.

We would like to thank the reviewer for their insightful summary and for recognizing the conceptual strength of our contribution.

We think the work is now suitable for publication. However, a small number of minor clarifications and refinements would further strengthen the manuscript and improve accessibility:

* Clarify whether enzymes map one-to-one to substrates or allow multi-substrate use; briefly note implications for lag dynamics.

We thank the reviewer for this insightful comment. We have updated the Methods section (lines 364-368) to explicitly state that our model assumes a one-to-one mapping between metabolic enzymes and substrates. We also clarified that this mapping is the mechanistic basis for our lag model, as the need to synthesize a new, specific enzyme pool is what generates the lag during resource switching.

* Add a short, main-text explanation linking ϕ to microbial physiology and its limitations.

We thank the reviewer for the suggestion. In the revised manuscript (lines 107-111), we have clarified that ϕ represents the basal proteomic investment in enzymes for resources that are not currently contributing to growth, including resources whose uptake is suppressed by regulatory mechanisms (in sequential utilizers) or resources that have been depleted (for both strategies). We also specify the fundamental physical limitation of this parameter: the total investment across all resources must not exceed the total metabolic proteome capacity ($n_R \phi \leq 1$).

* Briefly signpost early that growth rate is mechanistic, while biomass prevalence is the ecological outcome.

We have added a signpost in the Introduction (lines 64-67) to clarify the conceptual roles of our two metrics.

* Include a one-line summary that results are robust (and even strengthened) under constrained efficiencies.

We appreciate the reviewer's attention to this point. While we had discussed this effect in the original manuscript, we agree that a more prominent summary would improve clarity. We have now added an explicit one-line summary (line 272).

* Clarify more prominently that the mechanism relies on fluctuating environments and is unlikely in steady-state (e.g. chemostats).

We thank the reviewer for this important suggestion. We agree that the advantages we describe are intrinsically linked to the "boom-and-bust" nature of the environment. In the revised Discussion (lines 310-318), we have added a paragraph to explicitly distinguish our findings from steady-state systems like chemostats. We clarify that our proposed mechanisms rely on the temporal niches inherent to fluctuating environments. In steady-state regimes, where resource concentrations are invariant, these temporal niches do not manifest, and competition would likely be governed by a different set of physiological and ecological trade-offs, which is beyond the scope of the present work.

* Define “mature communities” at first use.

We apologize for the lack of clarity. While a parenthetical definition was included in the Abstract, we agree that it should be explicitly defined upon its first use in the main text. We have updated the Introduction (lines 72-73) to clearly state that "mature communities" refers

to those assembled from a larger species pool through a more extensive process of invasion and colonization attempts.
